# Identification of Oncolytic Avian Reovirus Receptors in B16-F10 Cells and the Signaling-Mediated Pathways Involved in Viral Entry

**DOI:** 10.3390/v18030350

**Published:** 2026-03-12

**Authors:** Chao-Yu Hsu, Bo-Yan Tu, Jyun-Yi Li, Tsai-Ling Liao, Yi-Ying Wu, Chia-Ying Lin, Yu-Kang Chang, Muhammad Munir, Hung-Jen Liu

**Affiliations:** 1 Division of Urology, Department of Surgery, Tungs’ Taichung MetroHarbor Hospital, Taichung 435, Taiwan; t4361@ms.sltung.com.tw; 2 Institute of Molecular Biology, National Chung Hsing University, Taichung 402, Taiwan; brian891008@gmail.com (B.-Y.T.); alert109209@hotmail.com (J.-Y.L.); yiying939@gmail.com (Y.-Y.W.); 3 The IEGG and Animal Biotechnology Center, National Chung Hsing University, Taichung 402, Taiwan; 4 Department of Medical Research, Taichung Veterans General Hospital, Taichung 407, Taiwan; tlliao1972@gmail.com; 5 Rong Hsing Research Center for Translational Medicine, National Chung Hsing University, Taichung 402, Taiwan; 6 Ph.D. Program in Translational Medicine, National Chung Hsing University, Taichung 402, Taiwan; 7 Department of Beauty Science, Meiho University, Pingtung 912, Taiwan; 8 Department of Medical Research, Tungs’ Taichung MetroHarbor Hospital, Taichung 435, Taiwan; 9 Division of Biomedical and Life Sciences, Faculty of Health and Medicine, Lancaster University, Lancashire LA1 4YW, UK; muhammad.munir@lancaster.ac.uk; 10 Department of Life Sciences, National Chung Hsing University, Taichung 402, Taiwan

**Keywords:** avian reovirus 1, σC protein, Plg-RKT receptor, oncolytic virus, virus entry

## Abstract

Avian reovirus (ARV) is a major poultry pathogen recently recognized for its potential as an oncolytic virus that selectively infects and kills cancer cells without harming healthy human cells. However, the receptors mediating ARV entry into cancer cells remain unclear. Using mouse melanoma B16-F10 cells as a model, this study identified ARV-binding receptor candidates through viral overlay protein binding assay (VOPBA), SDS-PAGE, and LC-MS/MS analysis. Plaque-forming assays (PFAs) evaluated viral replication efficiency, while co-immunoprecipitation (Co-IP) and proximity ligation assay (PLA) confirmed direct interactions between viral σC and host receptor proteins. Functional assays using shRNA knockdown and antibody blocking demonstrated that inhibition of Plg-RKT expression markedly reduced ARV infection. Western blot analysis revealed that ARV binding to Plg-RKT activates Src and p38 MAPK signaling pathways, which promote caveolin-1 phosphorylation and caveolae-mediated endocytosis. These findings identify Plg-RKT as a crucial receptor mediating ARV σC binding and entry into B16-F10 melanoma cells. Furthermore, activation of Src-p38 MAPK signaling was shown to be essential for viral internalization. This study elucidates the molecular mechanism underlying ARV entry into melanoma cells and provides valuable insight for improving the selectivity and therapeutic potential of ARV as an oncolytic virus.

## 1. Introduction

Avian reovirus (ARV) belongs to the Reoviridae family and the Orthoreovirus genus. It is a non-enveloped, double-stranded RNA virus with an icosahedral symmetry, and its genome consists of ten discrete RNA segments [1]. ARV is one of the most prevalent avian pathogens worldwide [2], causing severe economic losses in the poultry industry. Its primary hosts include various bird species such as chickens and turkeys. Although ARV is not zoonotic, it induces a wide range of clinical symptoms, including viral arthritis and tenosynovitis, growth retardation, hepatitis, myocarditis, pericardial effusion, malabsorption syndrome, and multiple lesions in the respiratory, gastrointestinal, and central nervous systems [3]. ARV infection is also frequently associated with immunosuppression and virus-induced apoptosis [4,5,6]. Previous research in our laboratory demonstrated that the σC protein of ARV can induce apoptosis in baby hamster kidney (BHK-21), African green monkey kidney (Vero) cells, and human adenocarcinoma gastric (AGS) cells [6,7]. Numerous studies have confirmed that ARV possesses strong tumor selectivity and oncolytic potential, indicating its promise as an anticancer agent [8,9,10,11]. The ARV nonstructural protein p17 inhibits nuclear pore complex formation, leading to the accumulation and activation of p53 in the nucleus, which in turn upregulates p21 and PTEN, while suppressing the PI3K/AKT/mTOR and ERK signaling pathways. This induces cell cycle arrest and autophagy, facilitating viral replication [12]. Recent findings from our laboratory further revealed that ARV efficiently infects several cancer cell lines, including non-small cell lung cancer (A549), mouse melanoma (B16-F10), and human cervical carcinoma (HeLa), but not normal human lung fibroblasts (HFL-1). These results demonstrate that ARV selectively infects and kills cancer cells, confirming its classification as an oncolytic virus [6,11,13,14]. Moreover, clinical studies using gastric cancer patient-derived cells showed that ARV can synergize with CD8^+^ cytotoxic T lymphocytes to induce immunogenic apoptosis in gastric cancer cells [6]. Our previous findings also demonstrated that ARV utilizes Annexin A2 (AnxA2) and adhesion G-protein-coupled receptor L2 (ADGRL2) as viral attachment receptors [15], subsequently activating the Src and p38 MAPK signaling pathways to trigger receptor-mediated endocytosis for viral entry [15,16].

Although our laboratory has confirmed that ARV can infect various cancer cell lines, the specific receptors mediating ARV attachment and entry into most cancer cells remain largely unidentified. B16-F10 cells which are a widely used mouse melanoma cell line known for their aggressive metastatic potential, making them valuable for cancer research. Therefore, this study aims to identify the ARV receptors on the surface of mouse melanoma B16-F10 cells and to elucidate the downstream signaling pathways and entry mechanisms triggered upon virus–receptor interaction. Understanding these virus–host interactions will provide valuable insights for the clinical application of ARV as a potential oncolytic agent, enabling the identification of receptor-based biomarkers and improving the safety and tumor selectivity of ARV-based oncolytic virotherapy.

## 2. Materials and Methods

### 2.1. Cell Lines

Cell lines used in this study included mouse melanoma cells (B16-F10), HFL-1, and African green monkey kidney (Vero) cells. B16-F10 were cultured in Dulbecco’s Modified Eagle Medium (DMEM), HFL-1 were cultured in Kaighn’s modification of Ham’s F-12 medium (F12K), and Vero cells were cultured in Minimum Essential Medium (MEM) with 5–10% fetal bovine serum (FBS) (Hyclone, Logan, UT, USA) and 1% penicillin (100 IU/mL)/streptomycin (100 g/mL) (Gibco, Grand Island, NE, USA). Cells were maintained in a humidified incubator at 37 °C with 5% CO_2_. Once the cells reached approximately 80% confluence, they were detached and sub-cultured.

### 2.2. ARV S1133 Strain

The ARV S1133 strain was used in this study. Vero cells were used as the host for viral propagation. Cells were cultured in 10 cm dishes and infected with ARV at a multiplicity of infection (MOI) of 10 when confluence reached 90%. After 2 h of adsorption, the inoculum was replaced with fresh medium, and incubation continued until an evident cytopathic effect (CPE) was observed. Cells and supernatant were harvested, subjected to three cycles of freezing and thawing, and centrifuged at 12,000× *g* for 10 min. The clarified supernatant was collected and designated as the viral stock.

### 2.3. Antibodies

Mouse anti σC antibody was produced by our laboratory. Mouse anti-β-actin antibody was purchased from Millipore (Billerica, MA, USA). Rabbit anti-Src, rabbit anti-p38 MAPK, rabbit anti-caveolin-1, rabbit anti-p-Src (Tyr416), rabbit anti-p-p38 MAPK (Thr180/Tyr182), and rabbit anti-p-caveolin-1 (Tyr14) antibodies were purchased from Cell Signaling Technology (Danvers, MA, USA). Rabbit anti-dynamin 2 and rabbit anti-Plg-RKT antibodies were purchased from Abcam (Cambridge, UK). The secondary antibodies goat anti-mouse IgG (H + L) HRP conjugate, Goat anti-rabbit IgG (H + L) HRP conjugate, Goat anti-mouse IgG (H + L) fluorescent, and Goat anti-rabbit IgG (H + L) fluorescent antibodies labeled with rhodamine were purchased from KPL (Gaithersburg, MD, USA).

### 2.4. Short Hairpin RNA (shRNA)

The Anxa1 shRNA gene, Anxa2 shRNA gene, Anxa5 shRNA gene, LPHN2 shRNA gene, RPSA shRNA gene, Bsg shRNA gene, Vamp2 shRNA gene, Tmem230 shRNA gene, PLGRKT shRNA gene, Src shRNA gene, and Mapk1 shRNA gene in pLKO vector were purchased from RNAi core facility (Academia Sinica, Taipei, China). shRNAs used in this study are shown in Table 1.

### 2.5. Inhibitors

A2ti (A2t high-affinity inhibitor) was purchased from Merck (Darmstadt, Germany). Bumetanide (SLC12A2 inhibitor), Fluorizoline (PHB1/PHB2 inhibitor), and KL-11743 (SLC2A3 inhibitor) were purchased from MedChemExpress (MCE, Monmouth Junction, NJ, USA).

### 2.6. Transient Transfection

Cell transfection was performed using FuGENE^®^ HD Transfection Reagent (Promega, Madison, WI, USA). Approximately 3 × 10^5^ cells were seeded per well in a 6-well plate and allowed to reach 70–80% confluence before transfection. DNA plasmids or shRNA constructs were introduced according to the manufacturer’s protocol. After 6 h, the transfection medium was replaced with fresh culture medium, and the cells were further incubated for subsequent analyses.

### 2.7. Cell Viability 3-(4,5-Dimethylthiazol-2-Yl)-2,5-Diphenyltetrazolium Bromide (MTT) Assay

Cell viability was determined using the MTT colorimetric assay. Cells (1 × 10^4^ per well) were seeded in 96-well plates and treated as indicated. MTT solution (0.5 mg/mL) was added to each well, followed by incubation at 37 °C for 4 h. After removing the supernatant, the formazan crystals were dissolved in DMSO, and absorbance was measured at 570 nm using a microplate reader (Thermo Fisher Scientific, Waltham, MA, USA).

### 2.8. Plaque Forming Assay (PFA)

To determine virus titers, Vero cells were seeded into 24-well plates at a density of 5 × 10^5^ cells per well and incubated at 37 °C in a humidified atmosphere containing 5% CO_2_ for 24 h until the cells reached approximately 90% confluence. The spent culture medium was then removed, and the cells were washed twice with 1× PBS. Serial 10-fold dilutions of the virus were prepared, and 500 μL per well of each viral dilution was added to the 24-well plates in order from the lowest to the highest viral concentration. The plates were subsequently incubated at 37 °C with 5% CO_2_ for an additional 24 h. After incubation, the virus-containing medium was removed, and 10 μL per well of trypan blue solution (Merck KGaA, Darmstadt, Germany) was added. The number of cytopathic effects (CPEs) was then counted under a microscope to calculate viral titers.

### 2.9. Cytoplasmic, Nuclear, and Membrane Protein Fractionation

Proteins from different cellular compartments were isolated using the CMNCS compartmental protein extraction kit (Biochain, Newark, NJ, USA). Cells designated for protein extraction were passaged, harvested, and collected into microcentrifuge tubes and removed the supernatant. Buffers C, N, and M were prepared according to the manufacturer’s instructions by adding 2% (*v*/*v*) of 50× protease inhibitor cocktail to each buffer, followed by gentle mixing, and kept on ice until use. Cell pellet was resuspended in buffer C and incubated with gentle agitation at 4 °C for 20 min. To disrupt the plasma membrane, a syringe fitted with a 26.5 G or larger needle, bent at approximately 30°, was used to repeatedly aspirate and expel the cell suspension. The lysate was then centrifuged at 12,000× *g* for 20 min at 4 °C, and the supernatant was collected as the cytoplasmic protein fraction. The remaining cell debris was resuspended in pre-chilled buffer W and incubated with gentle agitation at 4 °C for 5 min, followed by centrifugation at 12,000× *g* for 20 min at 4 °C. After discarding the supernatant, buffer N was added, and the sample was incubated at 4 °C for 20 min, then centrifuged again at 12,000× *g* for 20 min at 4 °C. The resulting supernatant was collected as the nuclear protein fraction. Finally, buffer M was added to the remaining pellet and incubated with gentle agitation at 4 °C for 20 min, followed by centrifugation at 12,000× *g* for 20 min at 4 °C. The supernatant obtained from this step was collected as the membrane protein fraction.

### 2.10. Virus Overlay Protein Binding Assay (VOPBA)

Virus overlay protein binding assay was performed to identify which receptors on the cell membrane interact with the virus. Membrane proteins of the cells designated for analysis were extracted using a cytoplasmic/nuclear/membrane protein fractionation kit. Proteins were separated by SDS-PAGE and subsequently transferred onto PVDF membranes. The PVDF membranes were blocked with blocking buffer containing 5% non-fat dry milk in 0.1% TBST on a rocking platform at room temperature for 120 min. After blocking, membranes were washed twice with 0.1% TBST and then incubated in 5 mL of fresh blocking buffer containing ARV at a MOI of 100. The membranes were incubated on a rocking platform at 4 °C for 16 h. On the following day, membranes were washed three times with 0.1% TBST for 10 min each, and then incubated with a primary antibody against ARV σC protein (1:4000 dilution) at 4 °C for 16 h. After primary antibody incubation, membranes were washed eight times with 0.1% TBST for 15 min each, followed by incubation with a secondary antibody diluted 1:5000 in blocking buffer on a rocking platform at room temperature for 120 min. The membranes were then washed again eight times with 0.1% TBST for 15 min each. After drying, protein–virus interactions were visualized using chemiluminescence detection. Based on the chemiluminescent signals, the molecular weights of membrane proteins capable of binding the virus were estimated. Corresponding protein bands from SDSPAGE gels were subsequently excised and subjected to LC-MS/MS (Triple TOF 6600, SCIEX, Framingham, MA, USA) analysis for viral receptor protein identification.

### 2.11. Liquid Chromatography–Tandem Mass Spectrometry (LC-MS/MS)

Protein identification was performed by the Proteomics and Metabolomics Mass Spectrometry Core Facility at the Taiwan, Taichung, Biotechnology Center, National Chung Hsing University.

### 2.12. Co-Immunoprecipitation (Co-IP)

Cells designated for collection were passaged, harvested, and transferred into microcentrifuge tubes. Cells were seeded in 6 cm cell culture dishes with DMEM containing 5% FBS and incubated at 37 °C with 5% CO_2_ until cell confluence reached about 75%. The cells were centrifuged at 300× *g* for 15 min to remove the supernatant, then washed twice with phosphate-buffered saline (PBS) and lysed in 300 mL of 3-[(3-cholamidopropyl)-dimethylammonio]-1-propanesulfonate (CHAPS) lysis buffer (40 mM HEPES [pH 7.5], 1 mM EDTA, 10 mM glycerophosphate, 120 mM NaCl, 50 mM NaF, 10 mM pyrophosphate, and 0.3% CHAPS). The lysates were centrifuged at 12,000× *g* for 10 min at 4 °C, and the supernatants were collected for protein quantification. Co-immunoprecipitation was performed using the Catch and Release v2.0 kit (Millipore Corporation, Billerica, MA, USA) according to the manufacturer’s instructions. 1500 μg of cell lysates, 3 μg of antibody, and 10 μL of the affinity ligand provided with the kit were added to the column. The volume was adjusted to 500 μL with 1× wash buffer, mixed thoroughly, and incubated overnight at 4 °C with gentle agitation. Following incubation, the beads were then resuspended with ice-cold 1× wash buffer and centrifuged at 2000× *g* for 30 s at 4 °C; this washing step was repeated twice. Finally, 100 μL of denaturing elution buffer was added to the column to fully cover the beads and incubated on ice for 5 min. The column was centrifuged at 10,000× *g* for 2 min at 4 °C, and the eluate was collected as the final sample. Sample was immediately mixed with an equal volume of 5× protein sample dye and heated in boiling water for 30 min to prevent protein degradation.

### 2.13. Proximity Ligation Assay

Proximity ligation assay (PLA) was performed using the Duolink^®^ PLA Multicolor Reagent Pack (Sigma-Aldrich, St. Louis, MI, USA) according to the manufacturer’s instructions. After fixation, cells were washed twice with 1× PBS and permeabilized with 1 mL of PBS containing 0.01% Triton X-100 on a rocking platform at room temperature for 20 min. Cells were then washed twice with 1× PBS and blocked with 1 mL of BlockPRO™ Protein-Free Blocking Buffer (Visual Protein, Taipei, China) at room temperature for 60 min. Cells were incubated with primary antibodies diluted 1:300 in blocking buffer at 4 °C for 16 h. Following primary antibody incubation, cells were washed twice with 1× Wash Buffer A for 5 min each. After washing, the PLA probe solution was added and incubated at 37 °C for 1 h followed by incubation with the ligation solution at 37 °C for 30 min. After ligation, cells were washed twice with 1× Wash Buffer A for 2 min each. The polymerase amplification solution was subsequently added, and samples were incubated at 37 °C for 100 min in the dark. After amplification, cells were washed twice with 1× Wash Buffer B for 10 min each. Nuclear counterstaining was performed by incubating cells with DAPI solution (0.01× Wash Buffer B containing 0.01% DAPI) on a rocking platform at room temperature for 15 min, followed by six washes with 0.01× Wash Buffer B for 10 min each. The slides were air-dried, and 10 μL of fluorescence mounting medium was added to evenly cover the cells and sealed with nail polish stored at 4 °C. Image acquisition and slide observation were performed under identical settings. The acquired images were processed and merged using ZEN Microscopy Software 3.0 (ZEISS), and fluorescence intensity quantification was performed using ImageJ software version 1.54j (National Institutes of Health, Bethesda, MA, USA).

### 2.14. Western Blot Assays

Cells were cultured in 6-well plates for 24 h before plasmid transfection or virus infection. After treatment the culture medium was removed from the cell culture dishes, and the cells were washed twice with 1× PBS. Cells were resuspended in 1× lysis buffer, the lysates were centrifuged at 12,000× *g* for 10 min at 4 °C, and the supernatants were collected for protein quantification with Bio-Rad Protein assay (Bio-Rad, Hercules, CA, USA). An appropriate volume of 5× protein sample dye was added, and the protein samples were collected into microcentrifuge tubes. The samples were subsequently heated in boiling water for 30 min to denature proteins. The samples were separated by 10–15% SDS–polyacrylamide gel electrophoresis and subsequently transferred onto PVDF membranes for Western blot analysis. The expression levels of individual proteins were detected using the corresponding primary antibodies, followed by incubation with horseradish peroxidase (HRP)-conjugated secondary antibodies. The PVDF membrane was incubated with an appropriate volume of enhanced chemiluminescence (ECL) detection reagent (Amersham Pharmacia Biotech, New Territories, Hong Kong, China). After chemiluminescent activation, the PVDF membrane was placed into a chemiluminescence imaging system (ATTO, Amherst, MA, SA) for signal detection and documentation.

### 2.15. Coomassie Brilliant Blue Staining

After completion of SDS–PAGE, the stacking gel was removed, and the resolving gel was transferred to a staining container. Coomassie Brilliant Blue staining solution (0.35% Coomassie Blue R-250, 45% methanol, 10% acetic acid) was added to completely immerse the gel, followed by staining on a rocking platform at room temperature for 1 h. After staining, the gel was rinsed twice with distilled water to remove excess dye and then incubated in destaining solution (20% methanol, 10% acetic acid) on a rocking platform at room temperature. Once the gel background became completely clear, the gel was placed in a plastic bag with a small amount of distilled water and scanned using a multifunction printer/scanner (Epson M2170, Seiko Epson, Suwa, Japan).

### 2.16. Immunofluorescence Assay

The sterile 18 × 18 mm coverslips were placed into 6-well culture plates, and 1.5 × 10^6^ cells per well were seeded onto the coverslips. Cells were incubated at 37 °C for 18 h prior to subsequent experimental treatments. After treatments the cells were washed twice with 1× PBS, followed by fixation with 1 mL of 10% formaldehyde solution at room temperature for 30 min. After fixation, cells were washed twice with 1× PBS and then incubated with 1 mL of PBS containing 0.01% Triton X-100 on a rocking platform at room temperature for 20 min to permeabilize the cell membrane and facilitate antibody and dye penetration. The cells were subsequently washed twice with 1× PBS and blocked with 1 mL of BlockPRO™ Protein-Free Blocking Buffer (Visual Protein, Taipei, China) on a rocking platform at room temperature for 60 min. Then cells were incubated with the primary antibody diluted 1:300 in blocking buffer at 4 °C for 16 h. Following primary antibody incubation, cells were washed six times with 0.05% PBST (1× PBS containing 0.05% Tween-20), 15 min per wash. Cells were then incubated with a TRITC-conjugated secondary antibody diluted 1:300 in blocking buffer at 4 °C for 16 h. After removal of the secondary antibody, cells were washed three times with 1× PBST for 15 min each, followed by incubation with 4’,6-diamidino-2-phenylindole (DAPI) solution (0.05% PBST containing 0.01% DAPI) on a rocking platform at room temperature for 15 min. Cells were then washed six times with 0.05% PBST, 15 min per wash. Finally, the slides were air-dried on the bench, and 10 μL of fluorescence mounting medium was added to evenly cover the cells. Coverslips were placed cell-side down onto glass slides and sealed with nail polish. The slides were stored at 4 °C. Immunofluorescence images were acquired using an inverted fluorescence microscope (ZEISS Axio Observer 3, Thornwood, NY, USA). Image acquisition and slide observation were performed under identical settings. The acquired images were processed and merged using ZEN Microscopy Software 3.0 (ZEISS), and fluorescence intensity quantification was performed using ImageJ software.

### 2.17. Statistical Analysis

Statistical analyses were performed using Student’s t-test to evaluate the significance of differences between groups. All data are presented as the mean± standard deviation (SD) from three independent experiments. A *p* value of < 0.05 was considered statistically significant.

## 3. Results

### 3.1. VOPBA Identification of ARV σC-Binding Membrane Proteins in B16-F10 Cells

**Figure 1:**
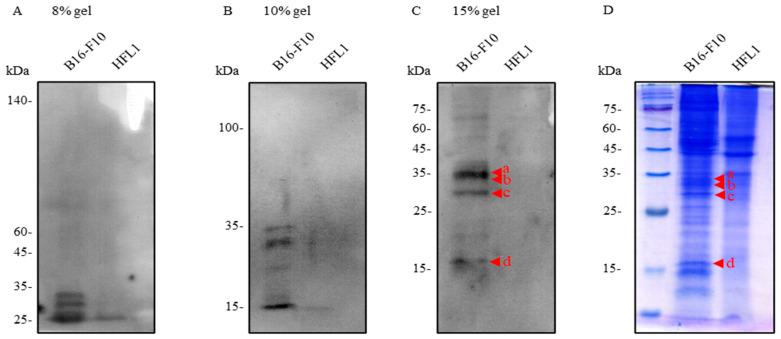
VOPBA-based identification of ARV σCbinding membrane proteins in B16-F10 cells. The isolated membrane protein samples of B16-F10 and HFL-1 cell lines were subjected to VOPBA using (**A**) 8%, (**B**) 10%, and (**C**) 15% polyacrylamide gels to analyze the distribution of B16-F10 membrane proteins capable of binding ARV σC across different molecular weight ranges. (**D**) The isolated membrane protein samples from B16-F10 and HFL-1 cell lines were separated by SDS-PAGE using 15% polyacrylamide gels and stained with Coomassie Brilliant Blue.

Previous studies from our laboratory have identified seven proteins as potential cellular receptors for ARV, including Annexin A1, Annexin A2, Annexin A5, and ADGRL2 [15]. Among these candidates, Annexin A2 and ADGRL2 have been validated as functional ARV receptors in Vero and DF-1 cells [15]. We have also demonstrated that the σC protein, located on the outer capsid of ARV, is capable of binding to cell surface receptors and activating downstream signaling pathways that facilitate viral entry into host cells [16]. To determine whether these cell surface proteins also serve as ARV receptors in B16-F10 cells, their expression was individually suppressed using shRNAs (Figure S1) or specific inhibitors (Figure S2), followed by infection with ARV MOI of 10 for 24 h and viral titers were measured using a plaque-forming assay. In addition, an MTT assay was performed to confirm that cell viability remained above 80% following shRNA transfection (Figure S3) or treatments with different concentrations of inhibitors (Figure S4). The results showed that none of the experimental groups exhibited a significant reduction in viral titers compared with the control groups, indicating that these seven proteins are unlikely to function as ARV receptors in B16-F10 cells. Therefore, additional candidate receptors must be identified to elucidate the mechanisms underlying ARV entry into B16-F10 cells. To identify potential ARV receptor proteins, virus overlay protein binding assay combined with LC-MS/MS analysis was employed to screen for cell membrane proteins capable of interacting with ARV σC. Membrane proteins were extracted from B16-F10 cells and HFL1 using a cytoplasmic/nuclear/membrane fractionation approach. HFL1 cells, which are not permissive to ARV infection, were included as a negative control. The extracted membrane proteins were subjected to VOPBA and SDS-PAGE using 8%, 10%, and 15% polyacrylamide gels, followed by LC-MS/MS-based protein identification. The results revealed four prominent protein bands at approximately 25–35 kDa and 15 kDa in the VOPBA analysis (Figure 1C a–d). Corresponding Coomassie brilliant blue-stained protein bands with relatively high expression levels were also observed at the same molecular weight positions in the SDS-PAGE gels (Figure 1D a–d). These four protein bands were excised from the gels and subjected to LC-MS/MS analysis for protein identification.

### 3.2. LC-MS/MS Identification of ARV-Binding Membrane Proteins

Liquid chromatography–tandem mass spectrometry is a powerful analytical technique that combines the physical separation capabilities of liquid chromatography with the highly sensitive and specific mass analysis of tandem mass spectrometry. Peptides are separated into a series of fragment ions providing a unique amino acid sequence fingerprint. Finally, bioinformatic algorithms, such as SEQUEST or MASCOT, compare these experimental spectra against theoretical spectra derived from protein databases to identify the original protein with high confidence [17]. The LC-MS/MS spectra were analyzed using the Matrix Science algorithm for spectrum comparison by optimal thresholding (MASCOT). Database searching was performed against the Swiss-Prot database, with the taxonomy restricted to Mus musculus. A total of 291 proteins were identified. To further narrow down potential ARV receptor candidates, the identified proteins were manually curated by consulting the Universal Protein Resource (UniProt) database and relevant literature. Proteins predicted or reported to be localized on the cell membrane, and those possessing transport functions, receptor activity, involvement in endocytosis-related cellular regulation, or previously reported as receptors for other viruses, were preferentially selected. Based on these criteria, 10 candidate proteins were identified as potential ARV receptors, among which five proteins had not been previously identified in our laboratory (Table 2).

### 3.3. Viral Titers and Intracellular σC Protein Expression Analysis

**Figure 2:**
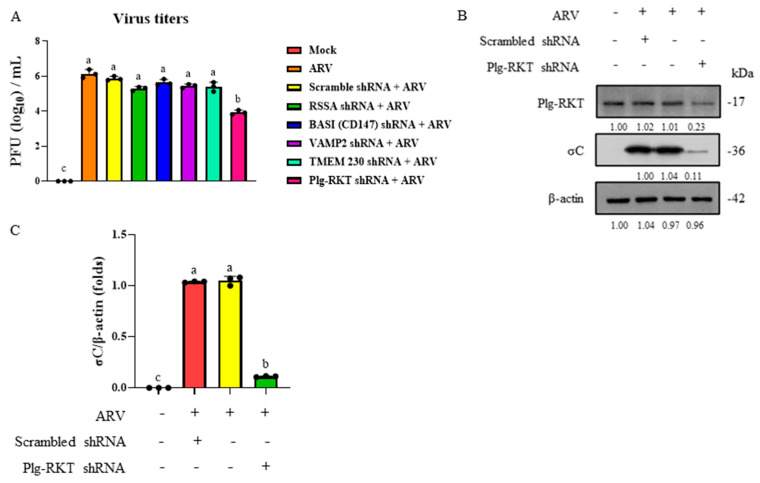
Viral titers and intracellular σC protein expression analysis. (**A**) B16-F10 cells were transfected with Scramble, RSSA, BASI, VAMP2, TMEM230, or Plg-RKT shRNA for 6 h, later infected with ARV at a MOI of 10 for 2 h. At 24 h post-infection, cells were subjected to repeated freeze–thaw cycles, and the supernatants were collected for viral titers analysis using PFA. (**B**) B16-F10 cells were transfected with Scramble or Plg-RKT shRNA for 6 h, later infected with ARV at a MOI of 10 for 24 h. Cell lysates were collected and analyzed by Western blot. Signals in Western blots were quantified with Image J. (**C**) Quantification was performed based on the σC protein expression shown in (**B**). All data are shown as the mean ± SD from three independent experiments. Statistical analysis was performed using Duncan’s multiple range test. Groups sharing the same letter designation (a, b, c) indicate no significant difference at *p* < 0.05.

In this study, the expression of the 5 new candidate proteins identified above was individually suppressed in B16-F10 cells using shRNA knockdown. The cells were then infected with ARV at a MOI of 10 for 2 h. At 24 h post-infection, the cells were subjected to repeated freeze–thaw cycles, and the supernatants were collected as viral samples. Changes in viral titers were subsequently determined using PFA. The results showed that suppression of Plg-RKT expression led to a greater than 100-fold reduction in viral titer compared with the control group (Figure 2A). In parallel, viral protein expression was analyzed by Western blot. The results demonstrated that inhibition of Plg-RKT by shRNA markedly reduced the expression level of the viral σC protein compared with the non-inhibited control group (Figure 2B,C). Collectively, these findings indicate that inhibition of Plg-RKT expression significantly suppresses ARV replication in B16-F10 cells, suggesting that Plg-RKT is a potential cellular receptor for ARV.

### 3.4. Analysis of Intracellular Viral σC Protein Expression by Antibody-Blocking Assay

**Figure 3:**
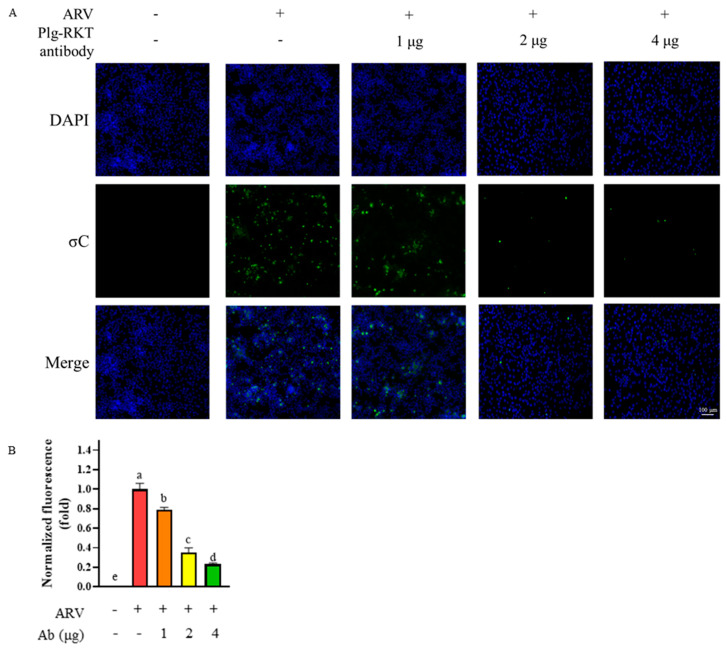
Analysis of intracellular viral σC protein expression by antibody-blocking assay. (**A**) B16-F10 cells were pretreated with 0, 1, 2, or 4 μg of anti-Plg-RKT antibody for 1 h to block cell surface Plg-RKT. Later infected with ARV at a MOI of 10 for 24 h. Fluorescence images were acquired using an inverted fluorescence microscope. Green fluorescence represents the viral σC protein, and blue fluorescence indicates DAPI-stained nuclei. (**B**) Fluorescence signals were quantified with ImageJ software. All data are presented as the mean ± SD from three independent experiments. Statistical analysis was performed using Duncan’s multiple range test. Groups sharing the same letter designation (a, b, c, d, e) indicate no significant difference at *p* < 0.05.

To further determine whether Plg-RKT is involved in ARV entry into B16-F10 cells, an antibody-blocking assay was performed. B16-F10 cells were pretreated with 0, 1, 2, or 4 μg of anti-Plg-RKT antibody for 1 h to block cell surface Plg-RKT. After removal of unbound antibodies, the cells were infected with ARV at a MOI of 10 for 2 h. Following infection, the cells were fixed and subjected to an immunofluorescence assay (IFA) to compare the intracellular fluorescence intensity of the viral σC protein between antibody-treated and untreated groups. The results showed that the fluorescence signal of σC decreased significantly in a dose-dependent manner with increasing concentrations of the anti-Plg-RKT antibody (Figure 3A,B). These findings indicate that Plg-RKT participates in ARV entry and infection of B16-F10 cells, supporting its role as a functional receptor involved in ARV infection.

### 3.5. Analysis of the Interaction Between ARV σC and the B16-F10 Cell Membrane Protein Plg-RKTs

**Figure 4:**
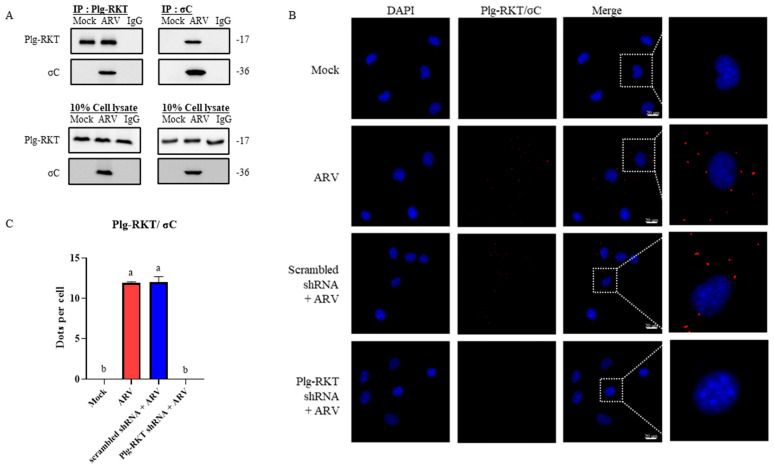
Analysis of the interaction between ARV σC and the B16-F10 cell membrane protein Plg-RKTs. (**A**) B16-F10 cells were infected with ARV (MOI = 10) for 24 h, followed by extraction of cell membrane proteins. Co-IP was performed using anti-Plg-RKT or anti-σC antibodies, precipitated samples were later analyzed by Western blot. IgG was used as a negative control. (**B**) PLA of ARV σC and Plg-RKT. B16-F10 cells were transfected with scrambled or Plg-RKT shRNA for 6 h, infected with ARV (MOI = 100) for 30 min, and subjected to PLA. Red fluorescence indicates σC–Plg-RKT interactions; blue fluorescence marks DAPI-stained nuclei. Magnified images correspond to the regions indicated by white boxes in the merged images. (**C**) Quantification was performed based on the fluorescent puncta shown in (**B**). Fluorescent signals were randomly quantified from 20 selected regions, with 100 cells analyzed in total. Data are presented as the mean ± SD. Statistical analysis was performed using Duncan’s multiple range test. Groups sharing the same letter designation (a, b) indicate no significant difference at *p* < 0.05.

To determine whether the ARV σC protein interacts with the cell surface membrane protein Plg-RKT in B16-F10 cells. B16-F10 cells were first infected with ARV at a MOI of 10 for 24 h. Cell membrane proteins were then extracted using a cytoplasmic/nuclear/membrane fractionation protocol. The resulting samples were subjected to co-immunoprecipitation later analysis by Western blot. The results showed that Plg-RKT and σC were co-immunoprecipitated, indicating that these two proteins can form a protein complex on the cell membrane, suggesting an indirect interaction between Plg-RKT and σC (Figure 4A). To further verify whether Plg-RKT and σC directly interact, a proximity ligation assay was performed. The principle of the PLA is based on the generation of a detectable fluorescent signal when two target proteins are located within 40 nm of each other, following circularization and amplification of conjugated DNA probes, thereby indicating a direct protein–protein interaction. Four experimental conditions were examined: Mock, ARV, Scrambled shRNA + ARV, and Plg-RKT shRNA + ARV. Cells were treated accordingly and infected with ARV at a MOI of 100 for 30 min, then later analysis by PLA. The results demonstrated the presence of a direct interaction between Plg-RKT and σC. In contrast, no fluorescent PLA signals were detected in cells that were not infected with ARV or in cells in which cell surface Plg-RKT expression was suppressed (Figure 4B,C). These findings indicate that Plg-RKT functions as an ARV receptor on B16-F10 cells by directly interacting with the viral σC protein.

### 3.6. Early ARV Infection Induces Phosphorylation of Src, p38 MAPK, and Caveolin-1 and Increases Dynamin-2 Expression in B16-F10 Cells

**Figure 5:**
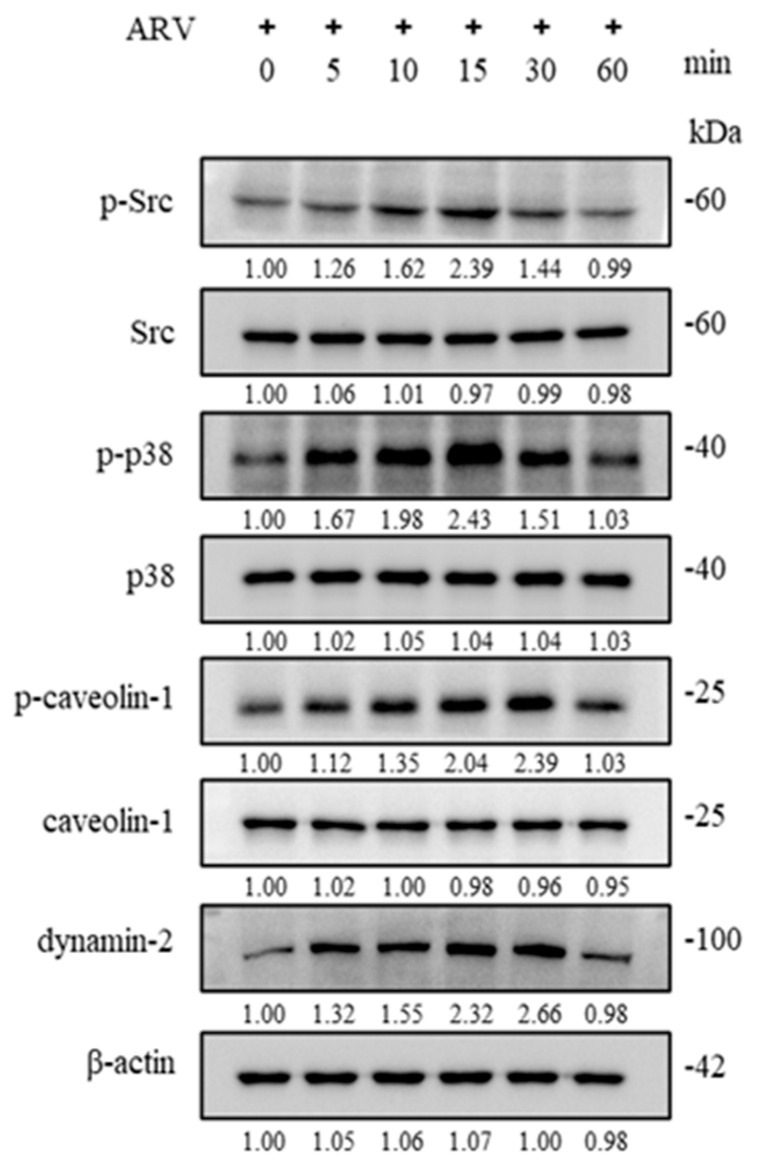
Western blot analysis of early ARV infection-induced changes in phosphorylated Src, p38 MAPK, caveolin-1, and dynamin-2 expression levels. B16-F10 cells were infected with ARV at a MOI of 10, and cell lysates were collected at 0, 5, 10, 15, 30, and 60 min post-infection. Changes in the expression levels of phosphorylated Src, p38 MAPK, caveolin-1, and dynamin-2 were analyzed by Western blot. Western blot signals were quantified using ImageJ software. Protein expression levels are presented as fold changes relative to the mock control. All data are shown as the mean ± SD from three independent experiments.

Previous studies from our laboratory have demonstrated that, following binding of ARV to cell surface receptors, the virus activates the Src and p38 MAPK signaling pathways, leading to downstream phosphorylation of caveolin-1 and upregulation of dynamin-2, thereby triggering endocytic uptake and facilitating viral entry into Vero cells and chicken embryo fibroblasts (DF-1) [15,16]. To determine whether ARV enters B16-F10 cells via a similar mechanism, B16-F10 cells were infected with ARV at a MOI of 10, and cell lysates were collected at 0, 5, 10, 15, 30, and 60 min post-infection. The activation status of the signaling pathway was then examined by Western blot analysis of phosphorylated Src, p38 MAPK, caveolin-1, and dynamin-2. The results showed that phosphorylated Src and p38 MAPK levels increased progressively after viral infection, reaching a peak at 15 min post-infection, followed by a gradual decline. Similarly, the expression levels of phosphorylated caveolin-1 and dynamin-2 increased in a time-dependent manner after ARV infection and reached maximal levels at 30 min post-infection, after which their levels decreased over time (Figure 5).

### 3.7. ARV Induces Activation of Src and p38 MAPK Signaling, Promoting Caveolin-1 Phosphorylation and Dynamin-2 Expression to Trigger Endocytosis in B16-F10 Cells

**Figure 6:**
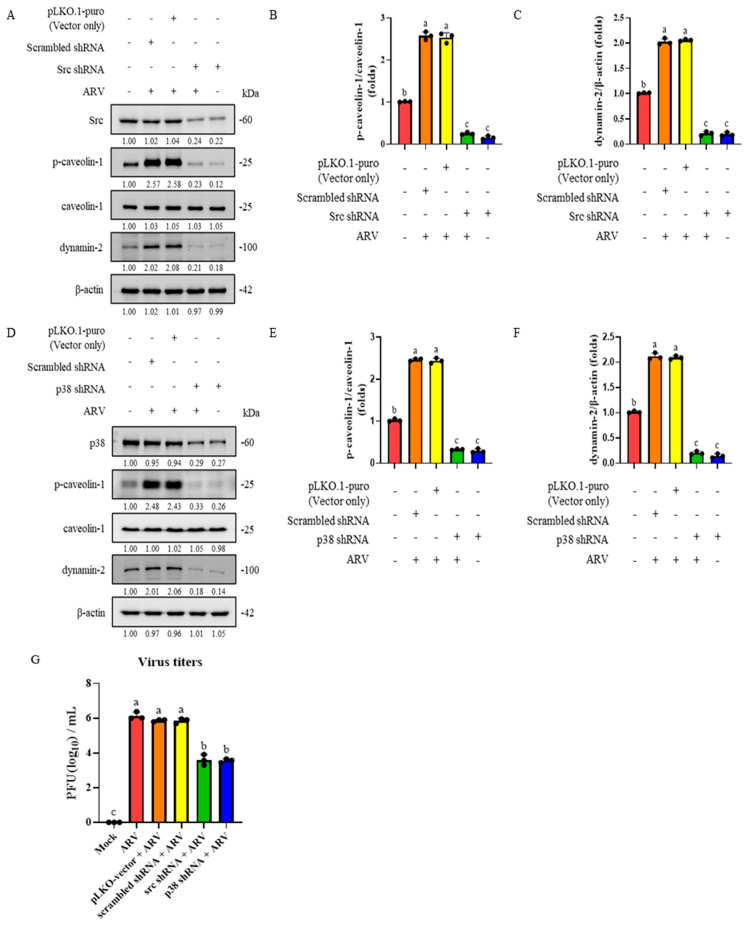
ARV induces activation of Src and p38 MAPK signaling, promoting caveolin-1 phosphorylation and dynamin-2 expression to trigger endocytosis in B16-F10 cells. (**A**) B16-F10 cells were transfected with Src shRNA, scrambled shRNA, or pLKO.1-puro for 6 h, followed by infection with ARV (MOI = 10) for 30 min. Quantification of the (**B**) phosphorylated caveolin-1 and (**C**) dynamin-2 expression levels shown in panel (**A**). (**D**) B16-F10 cells were transfected with p38 shRNA, scrambled shRNA, or pLKO.1-puro for 6 h, then infected with ARV (MOI = 10) for 30 min. Phosphorylated caveolin-1 and dynamin-2 levels were analyzed by Western blotting and quantified using ImageJ. Quantification of the (**E**) phosphorylated caveolin-1 and (**F**) dynamin-2 expression levels shown in panel (**D**). (**G**) B16-F10 cells were transfected with Src or p38 shRNA, scrambled shRNA, or the pLKO.1-puro vector for 6 h, followed by infection with ARV (MOI = 10) for 24 h. Viral titers were determined using PFA. Western blot signals were quantified using ImageJ and are presented as fold changes relative to the mock control. Data represent the mean ± SD from three independent experiments. Statistical analysis was conducted using Duncan’s multiple range test; groups sharing the same letter (a, b, c) indicate no significant difference at *p* < 0.05.

To further demonstrate that ARV enters cells through Src- and p38 MAPK-mediated signaling to promote caveolin-1-regulated endocytosis, we examined the effects of Src and p38 knockdown on downstream signaling events and viral replication. B16-F10 cells were transfected with Src or p38 shRNA for 6 h, later infected with ARV at a MOI of 10 for 30 min. Protein samples were then collected and analyzed by Western blot to assess the expression levels of phosphorylated caveolin-1 and dynamin-2. The results showed that knockdown of Src or p38 markedly reduced the levels of phosphorylated caveolin-1 and dynamin-2 expression compared with mock (Figure 6A,D). To further evaluate the impact of Src and p38 signaling on viral replication, B16-F10 cells were transfected with Src or p38 shRNA for 6 h and subsequently infected with ARV at a MOI of 10 for 24 h. Viral supernatants were collected after repeated freeze–thaw cycles, and viral titers were determined using PFA. The results demonstrated that suppression of Src or p38 significantly reduced intracellular viral titers (Figure 6G). Collectively, these findings indicate that activation of the Src and p38 MAPK signaling pathways is essential for caveolin-1 phosphorylation and dynamin-2 expression, and that this signaling cascade plays a critical role in ARV entry into host cells.

### 3.8. Suppression of Plg-RKT Expression Significantly Reduces ARV-Induced Phosphorylation of Src, p38 MAPK, and Caveolin-1, and Inhibits Dynamin-2 Expression

**Figure 7:**
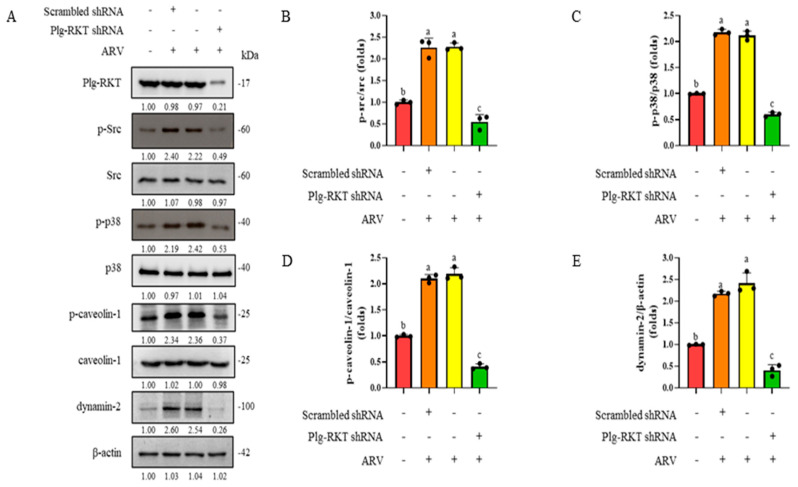
Suppression of Plg-RKT expression significantly reduces ARV-induced phosphorylation of Src, p38 MAPK, and caveolin-1, and inhibits dynamin-2 expression. (**A**) B16-F10 cells were transfected with Plg-RKT shRNA for 6 h, followed by infection with ARV at a MOI of 10 for 30 min. Protein samples were then collected and analyzed by Western blot. Quantification of (**B**) phosphorylated Src, (**C**) phosphorylated p38 MAPK, (**D**) phosphorylated caveolin-1, and (**E**) dynamin-2 expression levels were performed using ImageJ software. Protein expression levels are presented as fold changes relative to the mock control. Data represent the mean ± SD from three independent experiments. Statistical analysis was conducted using Duncan’s multiple range test; groups sharing the same letter (a, b, c) indicate no significant difference at *p* < 0.05.

Based on the results described above, early ARV infection of B16-F10 cells involves viral binding to cell surface receptors, leading to activation of the Src and p38 MAPK signaling pathways, which in turn promotes endocytosis-mediated viral entry. To determine whether suppression of Plg-RKT affects ARV-induced activation of Src and p38 MAPK, as well as downstream caveolin-1 phosphorylation and dynamin-2 expression, B16-F10 cells were treated with Plg-RKT shRNA for 6 h, followed by infection with ARV at a MOI of 10 for 30 min. Protein samples were then collected and analyzed by Western blot to examine changes in the expression levels of phosphorylated Src, p38 MAPK, caveolin-1, and dynamin-2. The results demonstrated that knockdown of Plg-RKT significantly reduced ARV-induced phosphorylation of Src and p38 MAPK in B16-F10 cells. Concurrently, phosphorylation of caveolin-1 and expression of dynamin-2 were also markedly suppressed (Figure 7). These findings indicate that Plg-RKT is required for ARV-induced activation of the Src–p38 MAPK signaling cascade and its downstream endocytic machinery during early infection.

## 4. Discussion

Despite advances in medical technology, cancer remains the second leading cause of death worldwide, with rising incidence and mortality rates [18]. Although conventional treatments such as surgery, chemotherapy, and radiotherapy are clinically effective, they are often accompanied by high costs, severe side effects, and drug resistance [19]. Immune checkpoint inhibitors have improved treatment specificity and patient survival; however, their clinical application is still constrained by cost, autoimmune toxicity, and variable efficacy across tumor types and microenvironments [19]. Therefore, the development of novel and more selective cancer therapeutic strategies has become an urgent need. Oncolytic virus therapy represents a promising anticancer strategy that selectively infects through recognizing specific receptors overexpressed on tumor cell surfaces and lyses tumor cells while stimulating antitumor immune responses [20,21]. Following viral oncolysis, the release of tumor-associated antigens further enhances immune activation. Several oncolytic viruses have advanced into clinical trials, including Talimogene laherparepvec (T-VEC), the first FDA-approved OV for melanoma, and mammalian reovirus, which has reached phase III clinical evaluation for multiple cancers [20,22]. These advances highlight the substantial clinical potential of oncolytic virus therapy and its important role in future cancer treatment strategies. As the development of oncolytic virus therapy continues, biosafety considerations have increasingly become a major focus of both academic and clinical research [23]. Enhancing tumor selectivity while minimizing adverse effects on normal tissues remains one of the key challenges in this field. Avian reovirus, a non-zoonotic virus, providing an inherent biosafety advantage for therapeutic application and has been shown to selectively infect and kill cancer cells without affecting normal human cells [14]. However, the susceptibility of cancer cells to avian reovirus varies, suggesting a dependence on cell surface receptor expression, which remains poorly defined. Therefore, this study aimed to identify the receptor mediating avian reovirus entry into B16-F10 melanoma cells and to elucidate the associated entry signaling pathways, providing a mechanistic basis for optimizing avian reovirus-based oncolytic therapies.

Cancer cells sustain rapid proliferation and metastasis by upregulating transcriptional and translational programs, largely through activation of the PI3K/AKT, mTOR, and RAS/MAPK pathways, which enhance eIF4F complex formation and protein synthesis [24]. This dysregulated translation favors tumor progression and is exploited by oncolytic viruses, which selectively recognize overexpressed or aberrant membrane proteins on cancer cells to facilitate viral entry and infection. Oncolytic viruses utilize diverse entry mechanisms. Enveloped viruses often enter cells via receptor-mediated membrane fusion, as exemplified by HSV-1, which binds HVEM or nectin-1, and vaccinia virus, which interacts with cell surface glycosaminoglycans. In contrast, non-enveloped viruses predominantly depend on endocytic pathways for host cell entry. Mammalian reovirus initially binds to cell surface sialic acid, followed by interaction with the tight junction protein JAM-A, which induces endocytosis and facilitates viral internalization. Similarly, adenovirus utilizes multiple receptors, including CAR, CD46, and DSG2, to trigger endocytic uptake into host cells [25]. Although the entry mechanisms of non-enveloped viruses have not been fully elucidated, it is well established that virus–receptor interactions represent a critical initiating step that triggers endocytosis and subsequent infection.

Previous studies from our laboratory demonstrated that the outer capsid σC protein of avian reovirus binds to Annexin A2 and ADGRL2 (Latrophilin-2) on the surface of Vero cells [15]. Annexin A2, a calcium-dependent phospholipid-binding protein widely expressed on the cell surface, has been implicated in the entry processes of numerous viruses. Binding of ARV σC to Annexin A2 activates downstream Src and p38 MAPK signaling pathways, which subsequently induce phosphorylation of caveolin-1 and promote the formation of caveolae structures, leading to invagination of the virus–receptor complex at the plasma membrane. Following this step, the GTPase activity of dynamin-2 mediates scission of virus-containing vesicles from the plasma membrane, resulting in the formation of early endosomes. This internalization process is referred to as caveolae-mediated endocytosis [15]. Furthermore, our laboratory has demonstrated that suppression of Annexin A2 or Latrophilin-2 expression significantly reduces ARV entry efficiency due to impaired viral binding and failure to initiate endocytosis. Likewise, inhibition of Src or p38 MAPK signaling blocks downstream caveolin-1 phosphorylation and caveolae formation, reduces dynamin-2 activity, and ultimately prevents completion of viral internalization, leading to a marked decrease in ARV infection efficiency [15,16]. Taken together, these findings indicate that avian reovirus exploits its outer capsid σC protein to bind specific host cell surface receptors, enabling selective recognition and infection of cancer cells. Viral entry is primarily mediated through caveolin-1-dependent, dynamin-2-regulated endocytosis. While this mechanism shares common features with other oncolytic viruses, such as reliance on receptor engagement and host endocytic pathways, avian reovirus also exhibits distinct entry strategies, highlighting the diverse evolutionary adaptations employed by different oncolytic viruses to gain access to host cells.

Previous studies have shown that the initial step of viral infection typically requires specific interactions between viral particles and host cell surface receptors. To identify potential receptor molecules exploited by avian reovirus on B16-F10 melanoma cells, this study employed virus overlay protein binding assay combined with LC-MS/MS analysis, resulting in the identification of 291 candidate proteins capable of binding to the virus. Subsequent filtering revealed that 10 of these proteins were associated with the regulation of endocytic pathways. Among these candidates, plasminogen receptor Plg-RKT has been reported to be highly expressed in several highly metastatic tumors, including lung cancer, and breast cancer, and is closely associated with enhanced tumor cell migration and invasiveness [26]. Although the precise biological functions of Plg-RKT remain incompletely understood, previous studies have demonstrated that Plg-RKT participates in the endocytic recycling of extracellular lipoproteins, such as apolipoprotein (a) [27]. In the present study, PFA results showed that suppression of Plg-RKT expression led to a significant reduction in intracellular viral titers, indicating that Plg-RKT is likely involved in the early stage of ARV infection, particularly during viral entry. Furthermore, antibody-blocking assays confirmed that masking Plg-RKT markedly decreased the efficiency of ARV entry into cells.

Importantly, co-immunoprecipitation and proximity ligation assay analyses demonstrated a direct interaction between the ARV σC protein and Plg-RKT, providing strong molecular evidence that Plg-RKT functions as a cell surface receptor for ARV. To further investigate whether Plg-RKT facilitates viral entry through regulation of endocytic pathways, we suppressed Plg-RKT, as well as the upstream endocytic regulators Src and p38 MAPK. The results revealed that ARV must first bind to Plg-RKT to activate the Src/p38 MAPK signaling cascade, which subsequently induces caveolin-1 phosphorylation and dynamin-2 expression, thereby completing the viral entry process. Although this study identifies Plg-RKT as a functional receptor for ARV in B16-F10 cells, it is unlikely to be the sole receptor involved. Notably, suppression of Plg-RKT reduced viral titers by more than 100-fold, yet residual ARV infection was still detectable. This observation suggests that while viral entry efficiency is substantially impaired, it is not completely abolished, indicating that ARV may utilize multiple receptors or co-receptors to enter host cells. Previous studies have shown that many viruses do not rely on a single receptor but instead employ a more complex, multi-receptor entry mechanism. It is well established that numerous viruses use at least two distinct types of receptors: a primary attachment receptor, which enables rapid viral binding to the cell surface, and a secondary or co-receptor, which facilitates subsequent steps such as stable attachment, internalization, and membrane fusion. Examples of such multi-receptor usage have been described for HIV-1, HSV-1 and HSV-2, adenovirus, and measles virus [28]. Therefore, Plg-RKT identified in this study is likely one component of a larger ARV receptor complex on B16-F10 cells. Further investigation is required to determine whether additional receptors or accessory molecules cooperate with Plg-RKT to regulate avian reovirus entry into B16-F10 melanoma cells, and to fully elucidate the molecular mechanisms governing this process.

## Figures and Tables

**Table 1:** shRNAs used in this study.

Target Gene	Cat. No.	Target Sequence (5′–3′)	Company
Anxa1	TRCN0000109728	GCACAAAGCTATCATGGTTAA	Academia Sinica, Taipei, China
Anxa2	TRCN0000110696	GTATGATGCTTCGGAACTAAA	Academia Sinica, Taipei, China
Anxa5	TRCN0000110701	CGAGAAAGTATTGACCGAGAT	Academia Sinica, Taipei, China
LPHN2	TRCN0000238691	TACCGCACCGATACGTTAATA	Academia Sinica, Taipei, China
RPSA	TRCN0000104471	GCTATTGTTGCCATCGAGAAT	Academia Sinica, Taipei, China
Bsg	TRCN0000054861	GCAATCACCAATAGCACTGAA	Academia Sinica, Taipei, China
Vamp2	TRCN0000325597	CCTCAAGATGATGATCATCTT	Academia Sinica, Taipei, China
Tmem230	TRCN0000320170	GCATCATGTAAGAGTTGTGAA	Academia Sinica, Taipei, China
PLGRKT	TRCN0000309561	CTTTAGCAACTGGAGCACTAA	Academia Sinica, Taipei, China
Src	TRCN0000023596	GCGGCTGCAGATTGTCAATAA	Academia Sinica, Taipei, China
Mapk1	TRCN0000054730	GCTGAATCACATCCTGGGTAT	Academia Sinica, Taipei, China

**Table 2:** LC–MS/MS Identification of ARV-binding membrane proteins.

Observed	Mr.		Delta	Score	Excepted Value	Match Sequence
	Excepted	Calculated				
**ANXA1**						
350.0958	698.1770	698.4690	−0.2920	45	0.01	K.ALLALAK.G
455.2967	908.5788	907.4399	1.1389	49	0.0051	R.ALYEAGER.R
916.1398	915.1325	915.4848	−0.3523	43	0.025	K.ILVALCGGN.-
607.1707	1212.3268	1212.5259	−0.1990	44	0.017	K.DITSDTSGDFR.K
671.1239	1340.2332	1340.6208	−0.3876	48	0.009	K.DITSDTSGDFRK.A
**ANXA2**						
622.9030	1243.7914	1243.6156	0.1758	51	0.0041	R.TNQELQEINR.V
**ANXA5**						
494.2732	986.5318	986.5284	0.0035	63	0.00022	R.TPEELSAIK.Q
501.1669	1000.3192	1000.5917	−0.2724	55	0.0015	K.VLTEIIASR.T
**PHB1**						
512.2872	1022.5598	1022.4920	0.0678	46	0.01	K.EFTEAVEAK.Q
529.6366	1057.2586	1057.5152	−0.2566	55	0.0015	K.QVAQQEAER.A
531.2540	1060.4934	1060.5400	−0.0466	45	0.017	K.AAIISAEGDSK.A
531.8109	1061.6072	1061.4989	0.1083	73	2.4 × 10^−5^	R.QVSDDLTER.A
575.2050	1148.3954	1148.5826	−0.1871	79	7 × 10^−6^	R.FDAGELITQR.E
603.1732	1204.3318	1204.6452	−0.3133	55	0.0015	R.FVVEKAEQQK.K
466.4580	1396.3522	1395.8350	0.5171	44	0.02	R.ILFRPVASQLPR.I
730.8025	1459.5904	1459.6467	−0.0562	78	9 × 10^−6^	R.IYTSIGEDYDER.V
**PHB2**						
417.6401	833.2656	833.4647	−0.1990	42	0.033	R.AQFLVEK.A
450.1206	898.2266	898.5600	−0.3333	50	0.0038	R.AQVSLLIR.R
497.5535	993.0924	993.4767	−0.3843	65	0.00014	R.LGLDYEER.V
528.1641	1054.3136	1054.6611	−0.3474	43	0.024	R.AQVSLLIRR.E
529.3915	1056.7684	1056.5312	0.2372	45	0.016	K.QVAQQEAQR.A
589.4067	1176.7988	1176.6251	0.1737	62	0.00032	K.FNASQLITQR.A
608.3218	1214.6290	1214.6142	0.0148	86	1.4 × 10^−6^	K.IVQAEGEAEAAK.M
736.2299	1470.4452	1470.7678	−0.3225	87	0.00057	R.QKIVQAEGEAEAAK.M
491.1585	1470.4537	1470.7678	−0.3141	88	0.0054	R.QKIVQAEGEAEAAK.M
768.9232	1535.8318	1535.8017	0.0301	89	1.2 × 10^−5^	K.MLGEALSKNPGYIK.L
**RSSA**						
602.3799	1202.7452	1202.6408	0.1045	94	2.3 × 10^−7^	K.FAAATGATPIAGR.F
653.5229	1305.0312	1305.6387	−0.6075	54	0.002	R.YVDIAIPCNNK.G
**BASI**						
549.0497	1096.0848	1096.5513	−0.4664	45	0.014	R.SEINVEGPPR.I
**VAMP2**						
424.21	846.4055	846.4083	−0.0028	50	0.00075	K.LSELDDR.A
833.4057	1664.7968	1664.8006	−0.0037	63	1.60 × 10^−6^	R.ADALQAGASQFETSAAK.L
**TM230**						
544.8	1087.5855	1087.5873	−0.0018	41	0.00073	R.TNLATGLPSSK.V
**PLRKT**						
568.8113	1135.6081	1135.6125	−0.0044	35	0.0062	K.GLITFESLEK.A

## Data Availability

Data are contained within the article and Supplementary Materials.

## Supplementary Materials

The following supporting information can be downloaded at: https://www.mdpi.com/article/10.3390/v18030350/s1, Figure S1: Screening of candidate ARV receptor proteins in B16-F10 cells using shRNA knockdown; Figure S2: Screening of candidate ARV receptor proteins in B16-F10 cells using inhibitors; Figure S3: Cell viability assay following shRNA transfection; Figure S4: Cell viability assay of inhibitors treated B16-F10 cells.
