# Supplementary material for: Identification of Oncolytic Avian Reovirus Receptors in B16-F10 Cells and the Signaling-Mediated Pathways Involved in Viral Entry
Source: Viruses. 2026 Mar 12;18(3):350. doi: 10.3390/v18030350 (PMC13030767; doi:10.3390/v18030350)
Supplement: Supplementary file 1 [file viruses-18-00350-s001.zip › viruses-4182556-supplementary.pdf]

Supplementary Figures

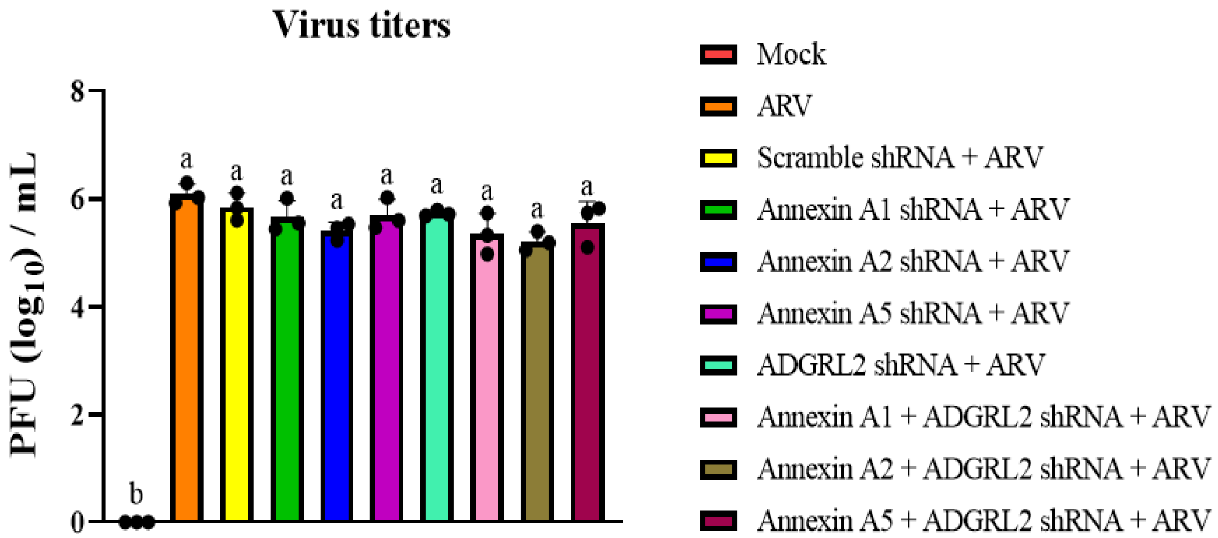

Supplementary Figure S1. Screening of candidate ARV receptor proteins in B16-F10 cells using shRNA knockdown. B16-F10 cells were cultured in 6-well plates until ~90% confluence and transfected with shRNAs targeting Annexin A1 (AnxA1), Annexin A2 (AnxA2), Annexin A5 (AnxA5), and ADGRL2, as well as combined knockdown groups (AnxA1 + ADGRL2, AnxA2 + ADGRL2, and AnxA5 + ADGRL2). A scramble shRNA was used as a negative control. At 6 h post-transfection, cells were infected with ARV at MOI of 10 for 2 h. At 24 h post-infection, viral supernatants were collected by three cycles of freeze–thaw, and viral titers were determined using a plaque-forming assay (PFA). Data are presented as mean ± SD from three independent experiments. Statistical analysis was performed using Duncan's multiple range test; groups labeled with the same letter (a, b) indicate no significant difference at $p < 0.05$.

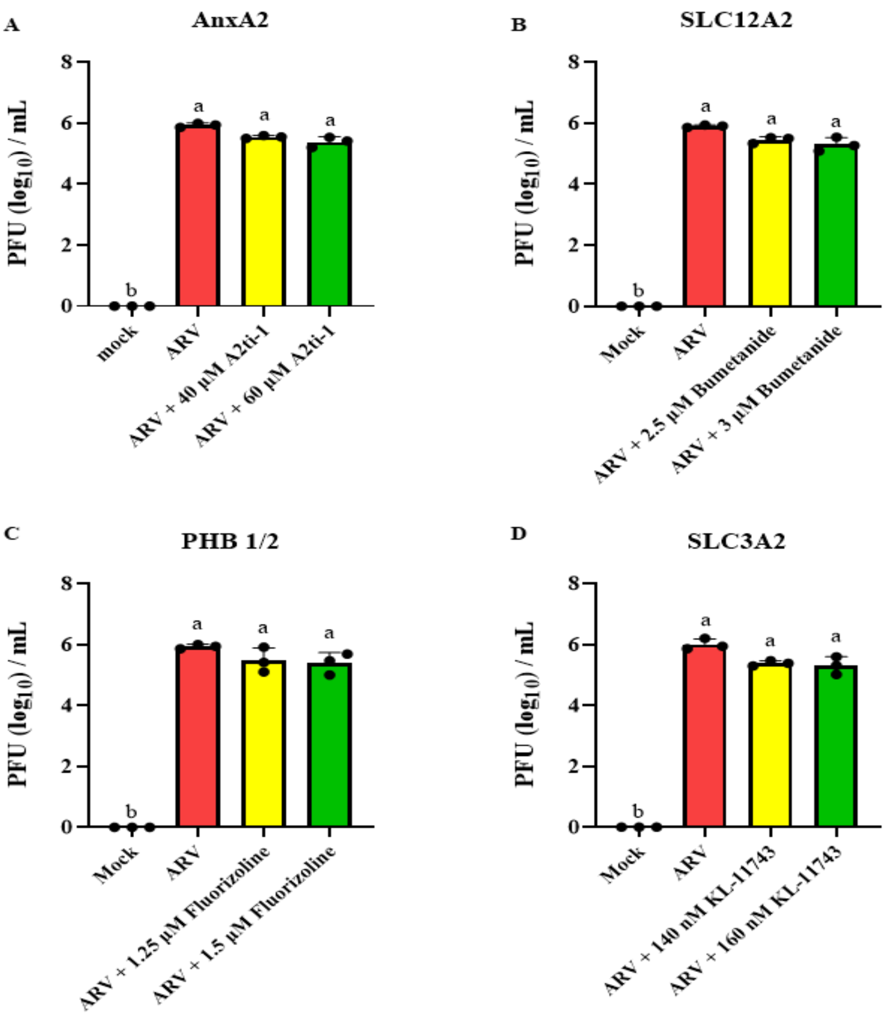

Supplementary Figure S2. Screening of candidate ARV receptor proteins in B16-F10 cells using inhibitors. (A) B16-F10 cells were treated with the Annexin A2 (AnxA2) inhibitor A2ti-1 at 40 µM or 60 µM for 6 h, followed by infection with ARV at MOI of 10 for 24 h. Viral titers were determined using a plaque-forming assay. (B) Cells were treated with the SLC12A2 inhibitor bumetanide at 2.5 µM or 3 µM for 6 h, infected with ARV at MOI of 10 for 24 h, and viral titers were analyzed by plaque-forming assay. (C) Cells were treated with the PHB1/2 inhibitor fluorizoline at 1.25 µM or 1.5 µM for 6 h, infected with ARV at MOI of 10 for 24 h, and viral titers were measured by plaque-forming assay. (D) Cells were treated with the AnxA2 inhibitor KL-11743 at 140 nM or 160 nM for 6 h, infected with ARV at MOI of 10 for 24 h, and viral titers were determined using plaque-forming assay. Data are presented as mean ± SD from three independent experiments. Statistical analysis was performed using Duncan's multiple range test; groups labeled with the same letter (a, b) indicate no significant difference at $p < 0.05$.

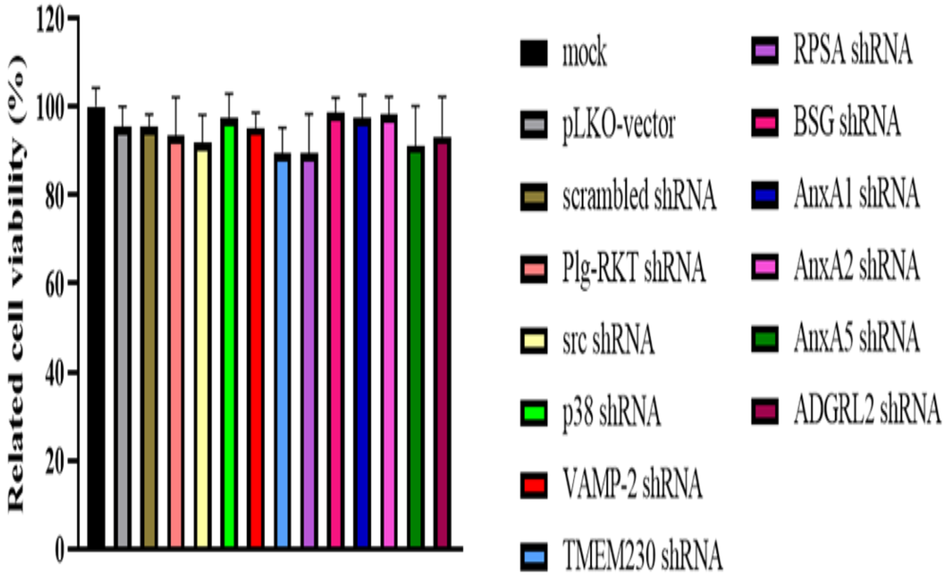

Supplementary Figure S3. Cell viability assay following shRNA transfection. B16-F10 cells were seeded in 96-well plates and transfected with the indicated shRNAs. At 6 h post-transfection, the transfection reagents were removed and replaced with fresh medium. After 24 h, cell viability was evaluated using the MTT assay to determine the effects of shRNA transfection on cellular viability. Data are presented as mean ± SD from three independent experiments.

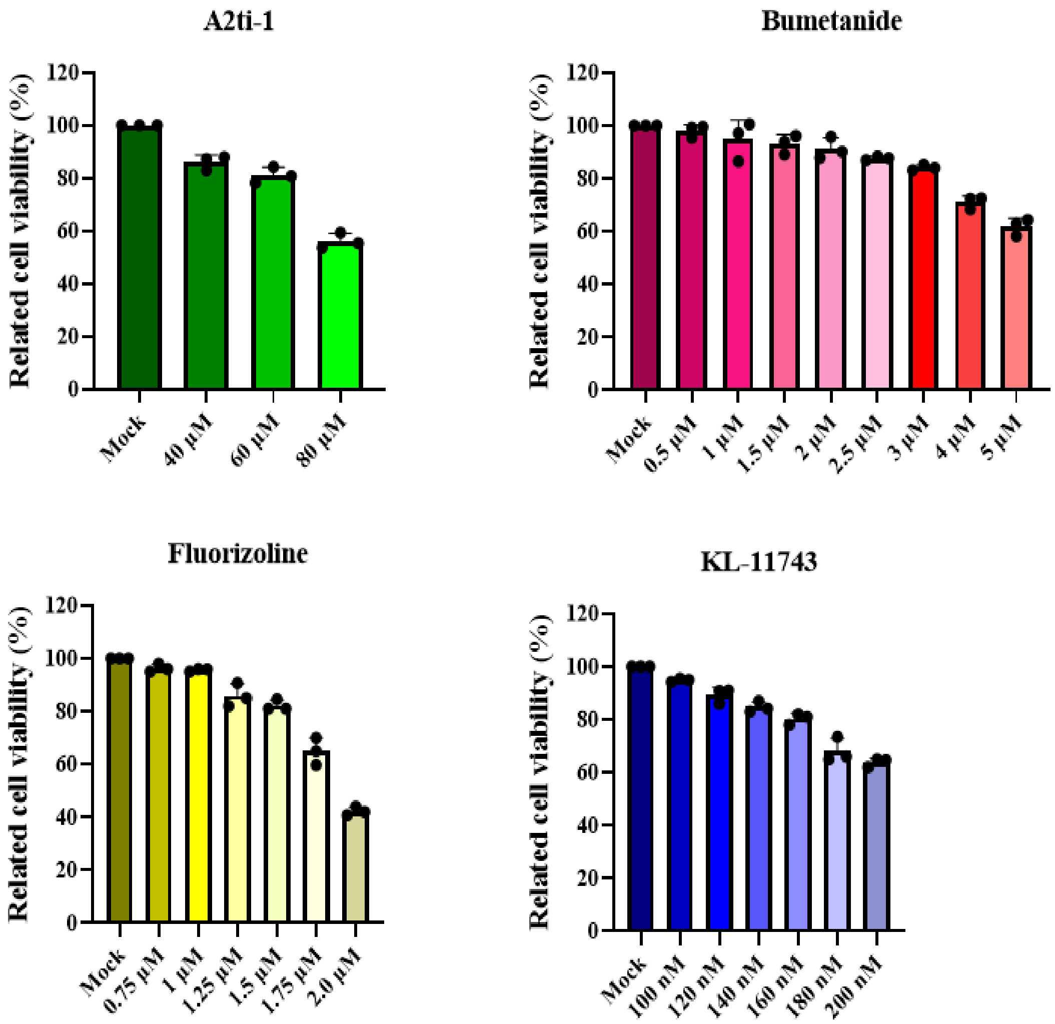

Supplementary Figure S4. Cell viability assay of inhibitors treated B16-F10 cells. B16-F10 cells were seeded in 96-well plates and treated with the indicated concentrations of inhibitors. AnxA2 was inhibited using A2ti-1, SLC12A2 was inhibited using bumetanide, PHB1/2 was inhibited using fluorizoline, and SLC2A3 was inhibited using KL-11743. After 24 h, cell viability was assessed to evaluate the cytotoxic effects of each inhibitor on B16-F10 cells. Data are presented as mean ± SD from three independent experiments.
